# Modulation of Gut Microbiota to Enhance Effect of Checkpoint Inhibitor Immunotherapy

**DOI:** 10.3389/fimmu.2021.669150

**Published:** 2021-06-29

**Authors:** Jianmin Wu, Shan Wang, Bo Zheng, Xinyao Qiu, Hongyang Wang, Lei Chen

**Affiliations:** ^1^ Institute of Metabolism & Integrative Biology (IMIB), Fudan University, Shanghai, China; ^2^ Department of Oncology, Shanghai Medical College, Fudan University Shanghai Cancer Center, Fudan University, Shanghai, China; ^3^ The International Cooperation Laboratory on Signal Transduction, Eastern Hepatobiliary Surgery Hospital, Second Military Medical University, Shanghai, China

**Keywords:** cancer treatment, tumor, immune checkpoint inhibitor, tumor immunotherapy, PD-1, CTLA-4

## Abstract

Accumulating evidence demonstrated the crucial role of gut microbiota in many human diseases, including cancer. Checkpoint inhibitor therapy has emerged as a novel treatment and has been clinically accepted as a major therapeutic strategy for cancer. Gut microbiota is related to cancer and the effect of immune checkpoint inhibitors (ICIs), and supplement with specific bacterial species can restore or enhance the responses to the ICIs. Namely, specified bacteria can serve as the biomarkers for distinguishing the patient who will respond to ICIs and determine the effectiveness of ICIs, as well as predicting the efficacy of checkpoint inhibitor immunotherapy. Regardless of the significant findings, the relationship between gut microbiota and the effect of ICIs treatment needs a more thorough understanding to provide more effective therapeutic plans and reduce treatment complication. In this review, we summarized the role of gut microbiota played in immune system and cancer. We mainly focus on the relationship between gut microbiota and the checkpoint inhibitor immunotherapy.

## Introduction

Microbes have been on earth for billions of years, they nearly occupy every corner of the earth, including the human body. The majority of microbes in our body resides in the gut (1). Gut microbiota consists of bacteria, fungi, virus, and other microbial and eukaryotic species and they mainly located in small intestinal and colon (1, 2). Gut microbiota colonized our body when we were born (3), and it has a great variety between different individuals (4). Based on previous research, gut microbiota has a close relation with various pathological conditions, including obesity (5), diabetes (6), neurodegenerative diseases (7), and cancers (8–11). Cancer is a major threat to human’s health with high fatality rate. At present, the major therapeutic strategy of cancer includes surgery, radiotherapy, chemotherapy, and immunotherapy (12). Cancer immunotherapy is a new therapeutic strategy emerging in recent years. Of note, cancer immunotherapy targeting immune checkpoints has achieved a great success. Antibody drugs targeting cytotoxic T-lymphocyte-associated protein 4 (CTLA-4) and programmed cell death protein 1 (PD-1) or its ligands programmed cell death protein ligand 1 (PD-L1) and 2 (PD-L2) arise in these years (13). The relevant antibody drugs like nivolumab, pembrolizumab were approved in clinical application by the United States Food and Drug Administration (FDA) (14). Most patients receiving ICIs treatment can achieve long-term suppression (13). But there still exist some limitations of ICIs, the response rates of patients treated with anti-PD-1 antibody are relatively low in several types of cancer (approximately 20 to 25% in non–small-cell lung cancer, melanoma, or renal-cell cancer) (15). What’s more, small number of patients gained hyperprogression after treating with ICIs (16), and approximately one third of patients relapse after treating with ICIs (13).

Recent studies reveal that the abundance and composition of gut microbiota can influence the effect of ICIs treatment (17). The relevant bacteria such as *Bifidobacterium* (18), *Bacteroides fragilis* (19), *Akkermansia muciniphia* (20) are found to be related to the clinical outcomes of cancer immunotherapy. Gut microbiota might serve as a potential factor affecting the effectiveness of checkpoint blockade immunotherapy. In this review, we summarized the role that gut microbiota played in immune system and in cancer genesis and development, we mainly focus on the relationship between gut microbiota and the effect of ICIs treatment.

## Gut Microbiota and Immune System

### Gut Microbiota and Innate Immunity

**Figure 1:**
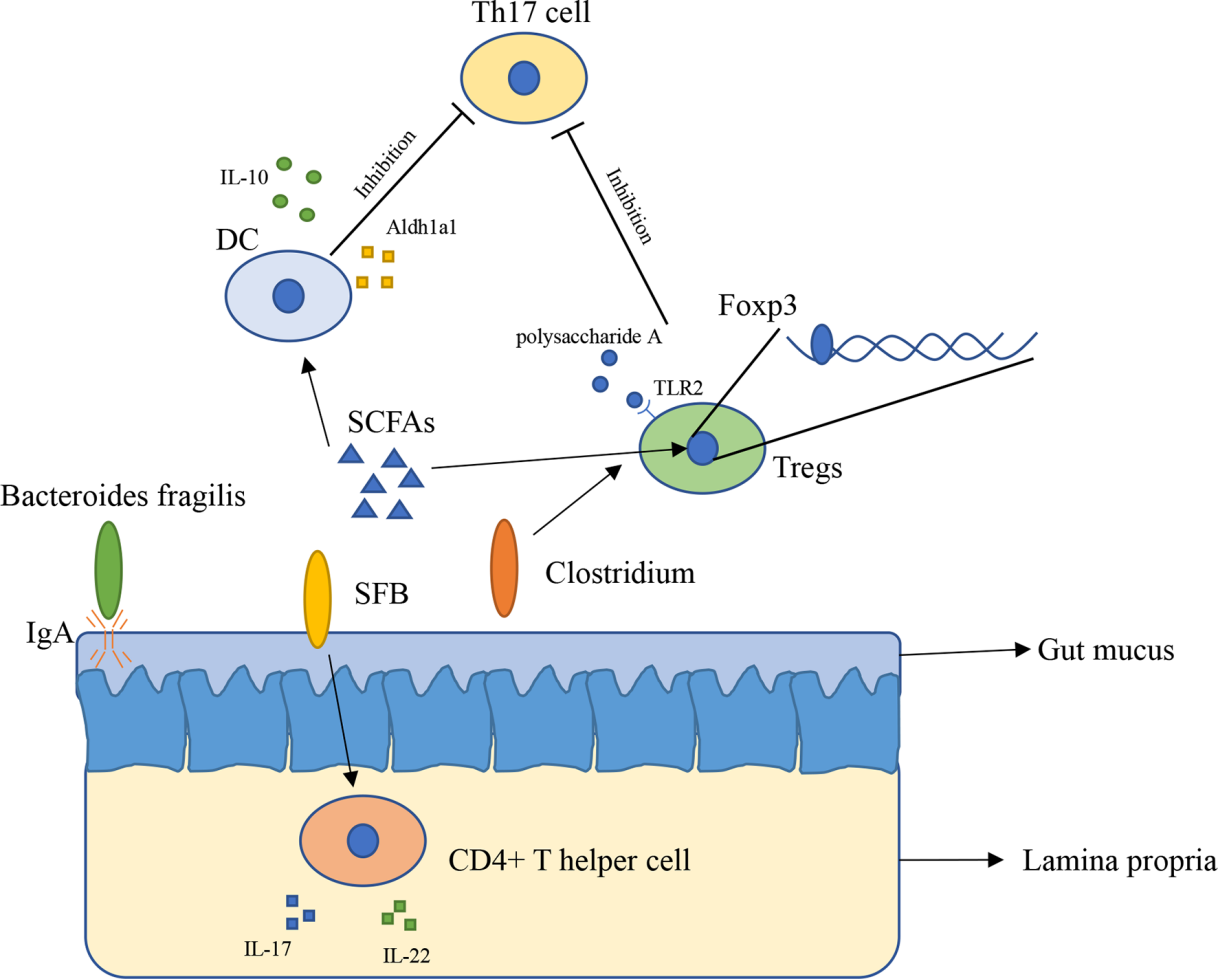
The interaction between gut microbiota and immune system (1). Bacteroides fragilis takes advantage of IgA to settle down in the intestine of mice (2); SFB induces the appearance of CD4+ T helper cells in lamina propria (3); Clusters IV and XIVa of the genus Clostridium are associated with Tregs accumulation in colon (4); SCFAs regulate Foxp3 in Tregs, inhibit immune response of T cells (5); SCFAs induce expressing of IL-10 and Aldh1a1 in DCs, result in inhibiting the development of Th17 cell (6); Polysaccharide A secreted by Bacteroides fragilis binds to TLR2 in Tregs, enhances Tregs and suppresses the proinflammatory Th17 response.

The innate immune system has physiological functions protecting our body. The innate immune system in intestinal consists of epithelial cells, myeloid cells, innate lymphoid cells, and other types of cells (21). Gut microbiota has broader effects on innate immunity (22). The immune system inside the intestinal plays a vital role in preventing external bacterial invasion and infection (22). Gut mucus is the first barrier providing the underlying epithelium from bacteria, to some extent, gut mucus is shaped by microbiota. The penetrability of gut mucus of germ-free mice and conventionally raised mice is different, the small intestine mucus become normally detached and colonic inner mucus become impenetrable after gavaged with microbiota from conventionally raised mice (23). In addition, immunoglobulin A (IgA) is an antibody isotype existing in intestinal lumen (24), mucosal IgA is binding to the polymeric immunoglobulin receptor and secreted across the epithelium (25), and it has a close relationship with *Bacteroides fragilis*. A research found that *Bacteroides fragilis* can take advantage of the immune system to settle down in the intestine of mice (**Figure 1**), however, it is difficult for *Bacteroides fragilis* to settle on the surface of the intestine and maintain long-term stability once the mice lack IgA, indicating that the immune system has a close relationship with commensal bacteria (26).

### Gut Microbiota and Adaptive Immunity

Gut microbiota has a close relation with adaptive immunity, especially T cells. A research found that in mice lamina propria, Th17 cells can be induced by *Segment filamentous bacteria* (SFB). Transplantation of SFB in mice induces the appearance of CD4+ T helper cells, increases level of IL-17 and IL-22 in lamina propria (**Figure 1**) (27). This research reveals that certain bacteria can cause changes in the number of specific cells in the immune system and expression of immune-related genes *in vitro*. However, how SFB affects Th17 cells remains unclear. Another research found that Indigenous *Clostridium* species promote the accumulation of CD4+ T regulatory cells (Tregs) in colonic mucosa. They found clusters IV and XIVa of the genus *Clostridium* is associated with Tregs accumulation in the colon (**Figure 1**) (28). Tregs plays an important role in maintenance of immune homeostasis, so this finding might offer a new therapeutic method to autoimmunity disease and allergy (28, 29). Gut microbiota influences immune system mainly through metabolites such as short-chain fatty acids (SCFAs). SCFAs are considered as the most abundant metabolites produced by gut microbiota in gut lumen, and they are produced by various types of bacteria including *Faecalibacterium prausnitzii*, *Roseburia intestinalis*, and *Anaerostipes butyraticus* (30, 31). SCFAs consist of acetate, propionate, and butyrate, they have an extensive influence on immune system. Studies have shown that butyrate can induce Treg cell differentiation *in vivo* or *in vitro* (32). Further research found that butyrate regulates Foxp3, which demonstrates that butyrate plays a role in inhibiting the activation and proliferation of T cells (32, 33). Butyrate also has an impact on Th17 cells through controlling the cytokine production by dentritic cells (DCs) (**Figure 1**). Butyrate produced by gut microbiota increases the level of GPR109a and induces expressing of IL-10 and Aldh1a1 in colonic macrophage and DCs, result in suppressing of development of Th17 cells (34). Feeding mice with *Bifidobacterium infantis* increased the production of retinoic acid in dendritic cells (DCs), result in increasing of TGF-β production and Treg expansion (35). Studies with gnotobiotic mice reveals an immunomodulatory effect of secreted microbial metabolites. In mice, *Bacteroides fragilis* secreted polysaccharide A binding to Toll-like receptor 2 (TLR2) on Treg cells, which can enhance the expansion of Tregs while suppressing the proinflammatory Th17 response (**Figure 1**), this indicated that polysaccharide A secreted by *Bacteroides fragilis* is able to prevent and cure experimental colitis in animals (36, 37). Through reading all of these researches, we can conclude that gut microbiota is indispensable for maintaining the immune homeostasis in animals and in human.

## Gut Microbiota and Cancer

### Gut Microbiota and Gastrointestinal Cancer

Gut microbiota has a great influence on gastrointestinal cancer (38, 39). The colonization of *Helicobacter pylori* (*H. pylori*) causes a persistent inflammatory response, which can lead to cancers of the stomach (38). Infected individuals with low abundance of *H. pylori* have a lower risk of gastric cancer, proving that *H. pylori* is associated with gastric carcinogenesis (40). Further studies revealed that *H. pylori* might promote cancer through β-catenin signal pathway (38). Colorectal cancer (CRC) is associated with specific bacteria including *Bacteroides fragilis*, *Fusobacterium nucleatum*, *Porphyromonas asaccharolytica*, *Parvimonas micra*, *Prevotella intermedia*, *Alistipes finegoldii*, and *Thermanaerovibrio acidaminovorans*, which can serve as potential diagnostic markers across population (41). Certain types of gut microbes might promote CRC. Sunny H. Wong et al. feed conventional and germ-free mice with stool samples from CRC patients and healthy individuals, then they found mice feeding with stool samples from CRC patients developed high-grade dysplasia (P <.05) and macroscopic polyps (P <.01) than mice feeding with stool samples from healthy individuals (42). Shaoguang W et al. found that Enterotoxigenic *Bacteroides fragilis* (ETBF) promotes colon tumorigenesis by stimulating immune response *via* Th17 cells in mouse CRC model, ETBF stimulates rapid colitis and colon tumors in multiple intestinal neoplasia (Min) mice and induce activation of transcription-3 (Stat3) in the colon of Min mice, Stat3 signaling is necessary for the generation of TH17 cells, and IL-17 produced by CD4+ Th17 is sufficient to induce tumorigenesis in the absence of γδ T cells (43). Gut microbiota also has a direct role in the occurrence of oncogenic mutations in colorectal cancer, a research reveals that colibactin—a compound generated by *Escherichia coli*—is believed to alkylate DNA on adenine residues and cause DNA double-strand breaks, which is related to the generation of colorectal cancer (44). Tumor promoting effects are mostly related to depletion or dysbiosis of gut-microbiota. After killing some bacteria with antibiotics (ATBs) in mice, the development of the tumor in the liver and the colon can be reduced (45, 46). Dianne H. Dapito et al. feed mice with ATBs which can eliminates commensal bacteria and reduce systemic lipopolysaccharide levels, then they can be found in DEN plus CCl4 HCC model, the tumor size was reduced (45).

### Gut Microbiota and Liver Cancer

Liver does not have direct contact with microbiota, but it has tight anatomic links to the gut (47, 48). In most of the studies, liver cirrhosis is a major potential risk of liver cancer (49). In 2016, people noticed that gut microbiota might associate with HCC progression in cirrhotic patients (50). Until now, there is no clinical evidence showing that a specific bacteria is associated with HCC (51). But experiments on gut-sterilized mice or germ-free mice showed that the development of HCC might associate with microbiota or microbially activated pathways, microbial metabolites, or microbiota-associated molecular patterns (MAMPs) also associate with the development of HCC (51). Gut microbiota and Toll-like receptors (TLRs) are required for HCC promotion but not required for HCC initiation (45). One research revealed that gut microbiota is associated with cholangiocarcinoma. According to the study which included 60 patients with cholangiocarcinoma, there existed a distinct, tissue-specific microbiome in bile duct tissues. Compared with normal samples (non-neoplastic liver), the patients with cholangiocarcinoma (CCA) tumors had a significant increase in *Stenotrophomonas* species in bile duct tissues (52).

### Gut Microbiota and Other Types of Cancer

There are also several studies focused on the gut microbiota and breast cancer. In 2015, Bard and his colleagues observed that gut microbiota was associated with clinical stages of breast cancer. They collected fecal samples from different stages of breast cancer patients, and performed 16S rRNA sequencing, they found that stage I patients had a lower absolute number of *Blautia* sp. than stage III, several other bacterial species (*Bifidobacterium*, *Blautia*, *F. Prausnitzii*, and *Blautia*) were different according to different clinical stages of breast cancer (53). Another research also found that gut microbiota was associated with different stages of breast cancer, compared to patients in stage 0/I, the number of *Bacteroidetes*, *Clostridium coccoides* cluster, *Clostridium leptum* cluster, *Faecalibacterium prausnitzii*, and *Blautia* sp. are increased in stage II/III patients. In addition, the absolute numbers of total bacteria are different according to the patient’s body mass index, which illustrated that obesity is associated with gut microbiota (54). Gut microbiota and lung has an essential cross-talk called “gut-lung axis” (55). In lung cancer patients, the level of *Enterococcus* sp. was decreased and the levels of *Actinobacteria* sp. and *Bifidobacterium* sp. were increased (56), indicating that lung cancer had an impact on the composition of gut microbiota.

### Metabolite of Gut Microbiota and Cancer

Metabolites of gut microbiota are known to induce proinflammatory cytokines and mediate tumor-associated inflammation in colon cancer (57, 58). The short chain fatty acids (SCFAs) secreted by bacteria (especially propionic acid and butyric acid) can mediate anti-inflammatory response and reduce the occurrence of colorectal cancer (59). Besides, SCFAs mediate p21 gene expression through modulation of microRNA, which will in turn influence colonic carcinogenesis (60). *Bacteroides fragilis* in the intestines of patients with colorectal cancer produce a variety of toxic metabolites such as β-glucuronidase, spermine oxidase, reactive oxygen species, reactive nitrogen species, and nitroso compounds, which can induce DNA damage and promote colorectal cancer (61, 62). Bile acid is an important component synthesized by the liver to regulate fat metabolism. Some anaerobic bacteria in intestine such as *Bacteroides* metabolize bile acids into secondary bile acids, which can cause DNA damage, stimulate EGFR or wnt/β-cantenin pathway, inducing colorectal cancer or liver cancer (46, 63, 64). Dietary or genetic obesity induces alterations of gut microbiota. Fed mice with high-fat diet resulting in the increase of Gram-positive bacteria (46). High-fat diet also increases the levels of bacterially generated deoxycholic acid (DCA). DCA is an intestinal bacterial metabolite which can cause DNA damage (65). Accumulating of DCA can facilitate obesity-associated HCC development in mice (46). In liver cancer, bile acid metabolism changes caused by gut microbiota regulates the expression of CXCL16 and recruits CXCL16-mediated natural killer T (NKT) cells, which could control the liver tumor growth (66). NKT cells can kill tumor cells in a CD1d-dependent manner (51). Recent study revealed that inosine—a purine nucleoside molecule produced by gut microbiota—can enhance cancer immunotherapy response in mice (67).

## Gut Microbiota Is Associated With the Effect of ICI Immunotherapy

### Bifidobacterium

**Figure 2:**
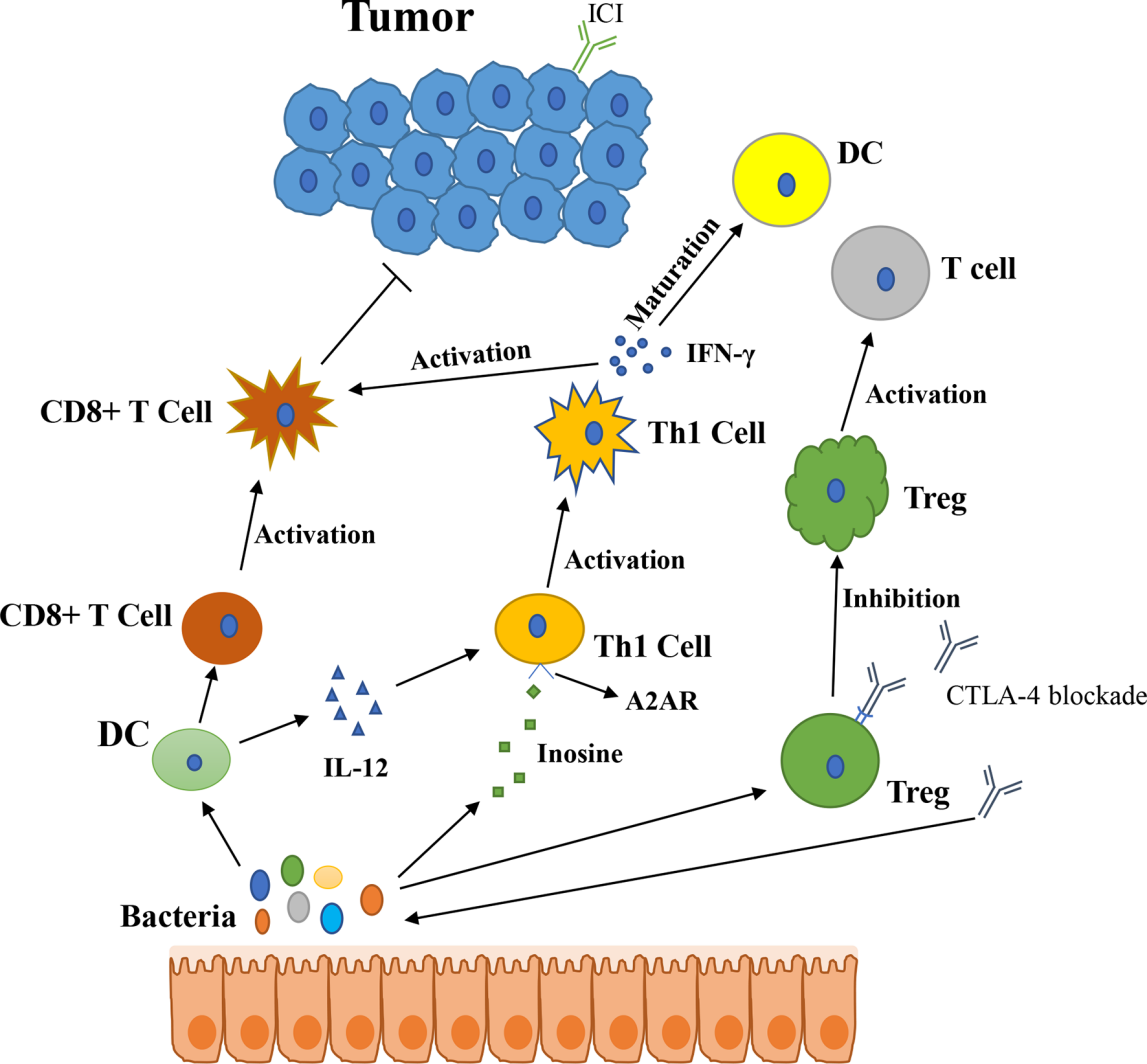
The potential mechanism of gut microbiota modulating the efficacy of ICIs (1). Bacteroides thetaiotaomicron or Bacteroides fragilis enhances T cell response, treatment of anti-CTLA-4 blockade influences the abundance of immunogenic Bacteroides spp., which will in turn enhance immune response of T cells (2); Bacteroides fragilis induces Th1 immune response, promotes the maturation of dendritic cell (3); *Bifidobacterium* improves function of DC, results in activating of CD8+ T cell and enhancing anti-PD-L1 antitumor effect (4); The metabolite inosine of gut microbiota activates Th1 cell and increases level of IFN-γ, which can enhance antitumor effect *in vivo* (5); Microbiota composition affects IL-12-independent Th1 cell immune response (6); CTLA-4 blockade induces the inactivation of CTLA-4+ Treg cells, leads to activating of effector T cell and enhancing antitumor effect.

In 2015, Thomas and his colleagues first noticed that there were correlations between gut microbiota and ICI immunotherapy (18). They used mice which were harbored with different commensal microbiota, then compared the melanoma growth of these mice. They also found that different microbiota might relate to different spontaneous antitumor immunity. Of which, they found that *Bifidobacterium* could facilitate antitumor effect of PD-L1 blockade (**Table 1**). Commensal *Bifidobacterium* controlled the growth of melanoma tumor in mice, oral administration of *Bifidobacterium* was an effective way to block tumor growth. *Bifidobacterium* treated mice showed a better antitumor effect compared to non-*Bifidobacterium* treated mice and this tumor control effect was related to tumor-specific T cells in periphery and accumulation of antigen-specific CD8+ T cells within the tumor (18). This study demonstrated that commensal *Bifidobacterium* can enhance antitumor immunity *in vivo* with an antigen-independent manner and it has a synergistic effect with PD-L1 blockade (18). In 2020, another team found that *Bifidobacterium* enhanced antitumor immunity through production of metabolite inosine (68). In this research, they found three bacterial species—*Bifidobacterium pseudolongum*, *Lactobacillus johnsonii*, and *Olsenella species*, these three bacterial species can significantly enhance the efficacy of ICIs in mouse models of CRC (**Table 1**) (68). They found that *Bifidobacterium pseudolongum* can enhance the antitumor effect through metabolite inosine, and this effect was dependent on adenosine 2A receptor (A_2A_R) signaling specifically in T cells. They also found that the antitumor effect of *Akkermansia muciniphia* in human also relies on inosine-A_2A_R signal (**Figure 2**). Recent research found that *Bifidobacterium* can enhance local anti-CD47 immunotherapy in tumor (71). The researchers used wild type (WT) mice, ATB-fed mice, and sterile mice to test the antitumor effects of CD47-based immunotherapy. The result showed that intestinal microbes outside the gastrointestinal tract are effective in promoting CD47 tumor immunotherapy. The results of *Bifidobacterium* injection experiments show that *Bifidobacterium* has antitumor effects in mice that do not respond to CD47 inhibition (71). This study reveals that the tumor-targeting ability of *Bifidobacterium* might be a possible mechanism that gut microbiota affect the antitumor response.

**Table 1:** Modulatory function of gut microbiome in ICIs therapy.

Bacteria	Model	Treatment	Cancer	Modulatory function in ICI therapy	Author/Year	Ref
*Bifidobacterium*	Mouse	PD-L1 blockade	Melanoma	a) Improve antitumor immunity	Ayelet Sivan 2015	(18)
				b) Enhancing dendritic cell function		
				c) Enhancing local anti-CD47 immunotherapy in tumor		
*Bifidobacterium pseudolongum*	Mouse	Immune checkpoint blockade	CRC	Increasing metabolite inosine production	Lukas F. Mager 2020	(68)
*Lactobacillus johnsonii*						
*Olsenella* species						
*Akkermansia muciniphia*	Human/Mouse	PD-1 blockade	Epithelial tumors	Enhancing the antitumor effect of PD-1 blockade	Bertrand Routy 2018	(20)
*Bacteroides fragilis*	Human/Mouse	CTLA-4 blockade	Melanoma/colon cancer	Influence interleukin 12 (IL-12)-dependent TH1 immune responses	Marie Vétizou 2015	(19)
*Bacteroides thetaiotaomicron*						
*Burkholderiales*						
*Uminococcaceae* family	Human/Mouse	PD-1 blockade	Melanoma	Promote the infiltration of CD8+ T cells in tumors	Gopalakrishnan V 2018	(17)
*Clostridiales* order						
*Faecalibacterium* genus						
Ratio of *Prevotella* and *Bacteroides*	Human	PD-1/PD-L1 blockade	Gastrointestinal cancer	Related to nucleoside and nucleotide biosynthesis, lipid biosynthesis, sugar metabolism, and fermentation to short-chain fatty acids	Zhi Peng 2020	(69)
*Bifidobacterium longum*, *Collinsella aerofaciens*, and *Enterococcus faecium*	Human	PD-L1 blockade	Melanoma	a) Decreasing regulatory T cell	Vyara Matson 2018	(70)
				b) Increasing Batf3 dendritic cells		
				c) Enhancing Th1 responses		

### Bacteroide

In 2015, another group published a research article focused on the gut microbiota and the treatment of CTLA-4 blockade (19). They find that the effect of CTLA-4 blockade treatment is related to distinct *Bacteroides* species (*Bacteroides fragilis* and/or *Bacteroides thetaiotaomicron* and *Burkholderiales*) (**Table 1**). If gut microbiota was removed or destroyed in mice, then CTLA-4 blockade has no effect on tumors. Transplant with *B. fragilis* can benefit for antitumor effect of CTLA-4 blockade. They found that *Bacteroides thetaiotaomicron* or *Bacteroides fragilis* cause T cell response, which is associated with antitumor efficacy of CTLA-4 blockade (19). Treatment of anti-CTLA-4 blockade also influence the abundance of immunogenic *Bacteroides* spp. in the gut, which would in turn affect the antitumor efficacy of ICIs. Further research revealed that CTLA-4 blockade influences the composition of microbiota, oral feeding of *Bacteroides fragilis* induce Th1 immune response, promoting the maturation of dendritic cell, which facilitate tumor control in mice and patients (**Figure 2**) (19). However, a research focused on the PD-1 immunotherapy of melanoma patients revealed that responders have a lower abundance of *Bacteroidales* than the non-responders (17). They collected 112 melanoma patients who were undergoing anti-PD-1 immunotherapy, then they examined the gut microbiome of these patients, and they found the diversity and composition of gut microbiota are different between the responders and non-responders: *Clostridiales* order and *Ruminococcaceae* family are enriched in responders while *Bacteroidales* order are enriched in non-responders. The abundance of *Bacteroidales* were positively related to the frequency of Treg cells and myeloid-derived suppressor cells (17). This study revealed that the microbiome composition might have an impact on anti-PD-1 immunotherapy, patients with favorable gut microbiome like *Ruminococcaceae* or *Faecalibacterium* might have an enhancing antitumor immune response, and this antitumor immune response was related to more antigen presentation in the periphery and improved effector T cell function in the tumor microenvironment. On the contrary, patients with a high abundance of *Bacteroidales* might have a limited response to anti-PD-1 immunotherapy (17).

### Akkermansia muciniphila

PD-1-based immunotherapy is also associated with gut microbiota. Analyzing of stool samples from renal cell carcinoma (RCC) and non-small cell lung cancer (NSCLC) patients after treating with PD-1 blockade revealed that clinical outcomes were associated with the abundance of *Akkermansia muciniphia*, oral supplementation with *Akkermansia muciniphia* to non-responders restored the efficacy of PD-1 blockade, which demonstrated that the *Akkermansia muciniphia* can enhance the effect of PD-1 inhibitor (**Table 1**) (20). They also found that treating with ATB in mice inhibited the antitumor effect of PD-1 blockade, which demonstrated that dysbiosis of gut microbiome system is another factor affecting results of ICIs (20). Fecal microbiota transplantation (FMT) from patients who respond to ICIs to mice can enhance the antitumor effect of PD-1 blockades in mice, while FMT from non-responders failed to do so. Oral supplementation with *Akkermansia muciniphia* to non-responders can enhance the efficacy of PD-1 blockade, besides, a higher incidence of *E. hirae* was found in responders of NSCLC patients than in non-responders (20). Another research consisting of 31 metastatic renal cell carcinoma (mRCC) patients also found that the abundance of *Akkermansia muciniphila* is positively correlated with the outcomes of checkpoint inhibitors. After treating with nivolumab or nivolumab plus ipilimumab, stool samples were collected and analyzed from mRCC patients, the result revealed that several bacteria are related to clinical benefit. They also found that a better treatment outcome is associated with high microbial diversity (72). The mechanism of *Akkermansia muciniphila* which promotes the effect of ICIs might be related to inosine (**Figure 2**), *Akkermansia muciniphila* can produce inosine *in vitro* in ICIs-treated tumors. Anti-CTLA-4 immunotherapy combined with monocolonization of *Akkermansia muciniphila* increased antitumor immunity, and this was associated with T cell expression of A_2A_R (68). In HCC patients, *Akkermansia muciniphila* increased in responders after PD-1 immunotherapy. Besides, *Ruminococcaceae* spp. and other 18 species of bacteria were also increased in responders. In non-responders, the level of *Proteobacteria* increased (73).

### *Faecalibacterium, Clostridiales*, and *Ruminococcaceae*

Gopalakrishnan V et al. examined gut microbiome of melanoma patients who were treating with anti-PD-1 immunotherapy, they found the composition and diversity of responders are different from those of non-responders. *Clostridiales* order and *Ruminococcaceae* family and *Faecalibacterium* genus were enriched in responders and *Bacteroidales* order was enriched in non-responders (17). The existence of *Ruminococcaceae* family, *Clostridiales* order, and *Faecalibacterium* genus can promote the infiltration of CD8+ T cells in tumors (**Table 1**). Patients rich in *Clostridiales*, *Faecalibacterium*, or *Ruminococcaceae* in gut have a higher frequency of CD4+ and CD8+ T cells. Besides, patients with high abundance of *Faecalibacterium* had better antigen processing and presenting ability and a relatively high density of immune cells compared to patients with high abundance of *Bacteroidales* in the gut (17).

### Prevotella/Bacteroides

Since gut microbiota is a whole system, a small change in one species of bacteria may have an impact on others. A recent research reveals that after immunotherapy, the ratio of *Prevotella* and *Bacteroides* will be elevated (**Table 1**) (69). In a research which included 74 patients with advanced-stage gastrointestinal cancer, after anti-PD-1/PD-L1 treatment, they observed an elevation of *Prevotella/Bacteroides* ratio in patients with a preferred response to anti-PD-1/PD-L1 treatment and a particular responder harboring a significantly higher abundance of *Prevotella*, *Ruminococcaceae*, and *Lachnospiraceae*. *Ruminococcaceae* is also associated with a favorable objective response rate in NSCLC after treatment of immune checkpoint inhibitors (74). Besides, gut bacteria which are capable of SCFAs, were positively associated with anti-PD-1/PD-L1 response (69).

### Composition of Gut Microbiota

As we discussed before, gut microbiota is a complex system. The alteration of whole system not just specific bacteria also plays an important role which can influence the effect of ICIs. Lisa Derosa et al. analyzed fecal samples from RCC patients treated with nivolumab, they found the patients who had used ATBs recently reduced objective response by 19% compared to the patients who had not used ATBs, and the composition of gut microbiota was significantly changed in these patients (75). This research indicates that ATBs shift the composition of gut microbiota, resulting in decreased efficacy of ICIs. Bertrand Routy et al. studied the impact of ATBs on NSCLC, RCC, and urothelial patients who were received anti PD-1/PD-L1 blockade, and they found that progression-free survival (PFS) and overall survival (OS) were significantly shorter in the ATB-treated group, demonstrating that dysbiosis has an impact on therapeutic efficacy of ICIs (20). Besides, alpha diversity is also important factor in the composition of gut microbiota. Gopalakrishnan V et al. analyzed fecal microbiome samples from 43 melanoma patients who were undergoing PD-1 blockade immunotherapy, and they found responding patients have a higher alpha diversity of bacteria than the non-responders, they also found that patients with higher alpha diversity in their fecal samples had significantly prolonged PFS than the patients in low alpha diversity (17).

## Ongoing Clinical Trials of Gut Microbiota in ICIs

Using bacteria as a new method for cancer therapy aroused people’s attention since bacteria were first applied in tumor therapy in 1991 (76). To date, there are three major ways in which the gut microbiota is used in tumor treatment: oral probiotics, diet intervention, and FMT. Several researches reported that probiotics have antitumor effects, especially in CRC. The antitumor effects of probiotics *Clostridium butyricum* and *Bacillus subtilis* on CRC progression had been proved in mice (77). A clinical research found that oral *Lactobacillus johnsonii* can significantly reduce the tumor recurrence rate of colon cancer patients after surgery (78). Another 12-year prospective clinical study showed that long-term intake of high-dose yogurt (containing *Streptococcus thermophilus* and *Lactobacillus bulgaricus*) has a significantly reduced risk of colorectal cancer (79). However, although probiotics were proved to be beneficial with antitumor effects, probiotic supplementation with ICIs might generate conflicting results (80). Probiotic use after ATBs could have opposite effect and delay restoration of the gut microbiota (81). One study focused on melanoma patients reported that the effect of immunotherapy might be negatively influenced by probiotics (82). Thus, it is necessary to find out the relation and inner mechanism between probiotics and the effect of ICIs in other kind of tumors. Altered diet can rapidly change the composition of gut microbiome (83). Which means it might be a simple and safe way to modulate gut microbiome in ICI patients (84). Patients with advanced bowel cancer intake inulin rich in oligofructose can enhance the effect of chemotherapy (85). Some studies are ongoing to investigate the association of dietary intervention with ICIs (NCT03700437, NCT03595540).

To date, FMT had been widely used in many diseases including cancer treatment. Previous animal experiments showed that fed mice with specific bacteria or FMT can enhance the sensitivity of immunotherapy. Thus, make several specific bacteria into medicine might be a new clinical adjuvant treatment of immunotherapy to cancer. FMT had been already applied in clinical trials, Robert and his colleagues successfully treated immune checkpoint inhibitors-associated colitis using FMT, transplant of fecal microbiota reconstruct the gut microbiome of patients and increase the proportion of regulatory T-cells in the colonic mucosa. This trial demonstrated that reshaping of gut microbiome might abrogate ICI-associated colitis (86). Recently, there were two studies using FMT to promote response in ICI-refractory patients (87, 88). A phase I clinical trial (NCT03353402) was performed in patients with anti-PD-1 refractory metastatic melanoma, in these patients, 3 of 10 patients were responded after intervention of FMT (88). Another research also focused on anti-PD-1 therapy in advanced melanoma, and they found that FMT and anti-PD-1 changed tumor microenvironment and gut microbiome which led to overcoming resistance of anti-PD-1 immunotherapy (87). In consideration of the relationship between the gut microbiota and ICIs, formulating a personalized treatment plan for patients based on the characteristics of the gut microbiota may become a new plan to optimize ICIs in tumor. Until now, several clinical trials are proceeding (**Table 2**). Most of clinical trials focused on the FMT combined with ICIs (NCT04163289, NCT04130763, NCT04130763, NCT04056026, NCT03341143, NCT04116775), a phase I trial (NCT03686202) using a new treatment approach named microbial ecosystem therapeutics (MET), which can also achieve FMT.

**Table 2:** Clinical trials on gut microbiota and ICIs.

NCT number	Condition or disease	ICIs	Interventions	Title	No. of enrolled patients	Location	Phase
NCT03700437	Non-small Cell Lung Cancer	Pembrolizumab	Dietary intervention Fast-Mimicking Diet (FMD)	Fasting-Mimicking Diet with Chemo-immunotherapy in Non-small Cell Lung Cancer (NSCLC)	40	USA	Not Applicable
NCT03595540	Cancer	Opdivo, Keytruda	FMD for 5 days	Fasting-Mimicking Diet in Patients Undergoing Active Cancer Treatment	60	Italy	Not Applicable
	Breast Cancer						
	Colorectal Cancer						
NCT03686202	All solid tumors	PD-1/PD-L1 blockade	MET-4	Feasibility Study of Microbial Ecosystem Therapeutics (MET-4) to Evaluate Effects of Fecal Microbiome in Patients on ImmunOtherapy (MET4-IO)	65	Canada	Phase I
NCT04163289	RCC	Nivolumab, Ipilimumab	FMT	Preventing Toxicity in Renal Cancer Patients Treated With Immunotherapy Using Fecal Microbiota Transplantation (PERFORM)	20	Canada	Phase I
NCT04130763	Gastrointestinal system cancer	PD-1 blockade	FMT	Fecal Microbiota Transplant (FMT) Capsule for Improving the Efficacy of Anti- PD-1	5	China	Phase I
NCT03353402	Melanoma	PD-1 blockade	FMT	Fecal Microbiota Transplantation (FMT) in Metastatic Melanoma Patients Who Failed Immunotherapy	40	Israel	Phase I
NCT04056026	Metastatic mesothelioma	Pembrolizumab	FMT	A Single Dose FMT Infusion as an Adjunct to Keytruda for Metastatic Mesothelioma	1	USA	Phase I
NCT04208958	Metastatic cancer, melanoma, gastroesophageal junction adenocarcinoma, colorectal cancer	PD-1 blockade	VE800, nivolumab, vancomycin oral capsule	Study of VE800 and Nivolumab in Patients With Selected Types of Advanced or Metastatic Cancer (Consortium-IO)	111	USA	Phase I/II
NCT03341143	Melanoma	Pembrolizumab	FMT	Fecal Microbiota Transplant (FMT) in Melanoma Patients	20	USA	Phase II
NCT04116775	Prostate cancer	Pembrolizumab	FMT	Fecal Microbiota Transplant and Pembrolizumab for Men With Metastatic Castration Resistant Prostate Cancer	32	USA	Phase II
NCT04136470	NSCLC, melanoma	Nivolumab, Ipilimumab, Atezolizumab	—	BioForte Technology for in Silico Identification of Candidates for a New Microbiome-based Therapeutics and Diagnostics	130	Poland	Discovery
NCT04169867	Melanoma	Nivolumab, Ipilimumab, Atezolizumab	—	Polish Microbiome Map	1160	Poland	Discovery
NCT04291755	NSCLC, colorectal cancer	Pembrolizumab	Pembrolizumab injection	Development and Analysis of a Stool Bank for Cancer Patients	100	USA	Discovery
NCT03353402	Melanoma	PD-1 blockade	FMT	Fecal Microbiota Transplantation (FMT) in Metastatic Melanoma Patients Who Failed Immunotherapy	40	Israel	Phase I

MET4, Microbial Ecosystem Therapeutics.

VE800: an orally administered live biotherapeutic product consisting of 11 distinct nonpathogenic, nontoxigenic, commensal bacterial strains manufactured under GMP conditions.

Several trials (NCT04208958, NCT03341143, NCT04116775) had preferable primary outcomes and moved to phase II trial. All of these clinical trials indicate that gut microbiota might be an auxiliary method for ICIs antitumor therapy.

## Conclusions and Future Directions

Both of the gut microbiota and the immune system are quite complex systems in our body, the currently proven relationship between them is only the tip of the iceberg. According to the existing research reports, the gut microbiota has a considerable relationship with the effect of tumor immunotherapy. We have summarized the abundance and composition changes of gut microbiota during immunotherapy of ICIs. Most of these bacteria are associated with immune system especially T cells. Some bacteria can significantly enhance the antitumor effect of ICIs, which may give us a hint that we can use gut microbiota as an aid to immunotherapy. In addition, specific intestinal bacteria can be used as a biomarker for immunotherapy. Although most of the mechanisms of antitumor effect of bacteria remain unknown, so the next step might be exploring the inner mechanism of gut microbiota and checkpoint inhibitor immunotherapy.

There still exist some limitations of previous research, we know that gut microbiota is a homeostatic ecosystem. Although regulating one certain flora can regulate the efficacy of ICIs immunotherapy, it also causes unbalance of the whole system, which might have some potential risks to human body. At present, the classification of intestinal flora is mostly done by 16s rRNA sequencing. 16s rRNA is highly conserved in many different kinds of bacteria, so the classification of flora based on 16s rRNA sequencing is relatively inaccurate. What’s more, present analysis was mainly based on the fecal sample, but the microbes in the fecal sample do not completely reflect the real ecosystem inside the intestine, so how to detect the dynamic change of gut microbiota after ICIs treatment in the human body remains a problem.

In conclusion, linking intestinal flora with tumor immunotherapy is a new attempt, and based on the existing research, the gut microbiota has a significant impact on the effect of tumor immunotherapy. Which prove that the various systems of the human body influence each other.

## Author Contributions

HW and LC are directors of this paper. HW is responsible for the main direction of this paper. LC designed the structure of this paper. JW was responsible for writing of major part of this paper. SW was responsible for proofreading of this paper. BZ was responsible for writing the abstract of this paper. XQ was responsible for writing the introduction of this paper. All authors contributed to the article and approved the submitted version.

## Funding

This work was supported by the National Research Program of China (2017YFA0505803, 2017YFC0908100), the State Key project for Infectious Diseases (2018ZX10732202-001,2018ZX10302207-004), and National Natural Science Foundation of China (81790633, 61922047, and 81902412).

## Conflict of Interest

The authors declare that the research was conducted in the absence of any commercial or financial relationships that could be construed as a potential conflict of interest.
